# Guided growth: mechanism and reversibility of modulation

**DOI:** 10.1007/s11832-016-0778-9

**Published:** 2016-11-08

**Authors:** Martin Gottliebsen, Juan Manuel Shiguetomi-Medina, Ole Rahbek, Bjarne Møller-Madsen

**Affiliations:** Department of Children’s Orthopaedics, Aarhus University Hospital, Aarhus, Denmark

**Keywords:** Guided growth, Growth plate, Children, Epiphysiodesis, Deformities, Leg length discrepancy

## Abstract

In paediatric orthopaedics, deformities and discrepancies in length of bones are key problems that commonly need to be addressed in daily practice. An understanding of the physiology behind developing bones is crucial for planning treatment. Modulation of the growing bone can be performed in a number of ways. Here, we discuss the principles and mechanisms behind the techniques. Historically, the first procedures were destructive in their mechanism but reversible techniques were later developed with stapling of the growth plate being the gold standard treatment for decades. It has historically been used for both angular deformities and control of overall bone length. Today, tension band plating has partially overtaken stapling but this technique also carries a risk of complications. The diverging screws in these implants are probably mainly useful for hemiepiphysiodesis. We also discuss new minimally invasive techniques that may become important in future clinical practice.

## Introduction

The term ‘guided growth' was introduced by Stevens when he reported on the use of hemiepiphysiodesis with tension band plating technique to correct deformities in growing children [1, 2]. However, manipulation of the growing bone is an old concept that extends back to the origin of orthopaedics. In 1741, Andry published his book on prevention of deformities in children in Paris [3].

The creation of skeletal structures is a complex process which would normally lead to the child having bones of correct length and proportion with symmetry between the two sides of the body at skeletal maturity.

The physis or the growth plate is located at both ends of the long bones and is interposed between the epiphysis and the metaphysis. No significant blood vessels pass through the physis, and chondrocytes are embedded in extracellular matrix. This highly specialised cartilage tissue can be divided into three different cellular layers with distinct characteristics. The zone of reserve is situated below the epiphysis and consists of resting cells that can be moved to the zone of proliferation [4]. Cells are arranged in columns and multiply in the zone of proliferation. Matrix synthesis is started and is needed for the process that ensures longitudinal bone growth. In the zone of hypertrophy, cells enlarge but no more proliferation takes place. The cells continue to be orientated in columns and contribute to the bony elongation process. The most mature layers begin the mineralisation process and the cells undergo apoptosis. Metaphyseal blood vessels invade the area. New calcified septae are formed where osteoblasts can start synthesising bone matrix and create trabecular bone. Because matrix synthesis, cell division and enlargement takes place in the zone of proliferation and zone of hypertrophy, growth is directed away from the zone of reserve towards the metaphysis. A separate vascular invasion occurs in each end of the long bones and leads to the formation of secondary centres of ossification in the epiphyses. The physis contributes not only to longitudinal bone growth but also to diametrical growth of the bone. Specialised cartilage tissue surrounding the physis called the zone of Ranvier is the mechanism behind this. Transverse growth also takes place in the periosteum by a process similar to intramembranous ossification.

The process of regulating skeletal growth consists of genetic, hormonal, nutritional and environmental factors and remains only partially understood. Physical activity appears to be of less significance on the final size of the individual. Mechanical factors within normal range seem to have a limited effect relative to genetic, nutritional and hormonal controls. Variations in the degree of cell hypertrophy probably contribute to a large part of the differences in growth rate. This process is apparently regulated hormonally [5]. The periosteal sleeve is inelastic and tends to resist growth. Could the periosteal sleeve be involved in determining growth rate? Circumcision of the metaphyseal sleeve can be used to stimulate longitudinal bone growth although the outcome is unpredictable. It is a well-described phenomenon that a fracture with impaired periosteal healing can lead to overgrowth of the affected side.

## Manipulation of skeletal growth

**Fig. 1:**
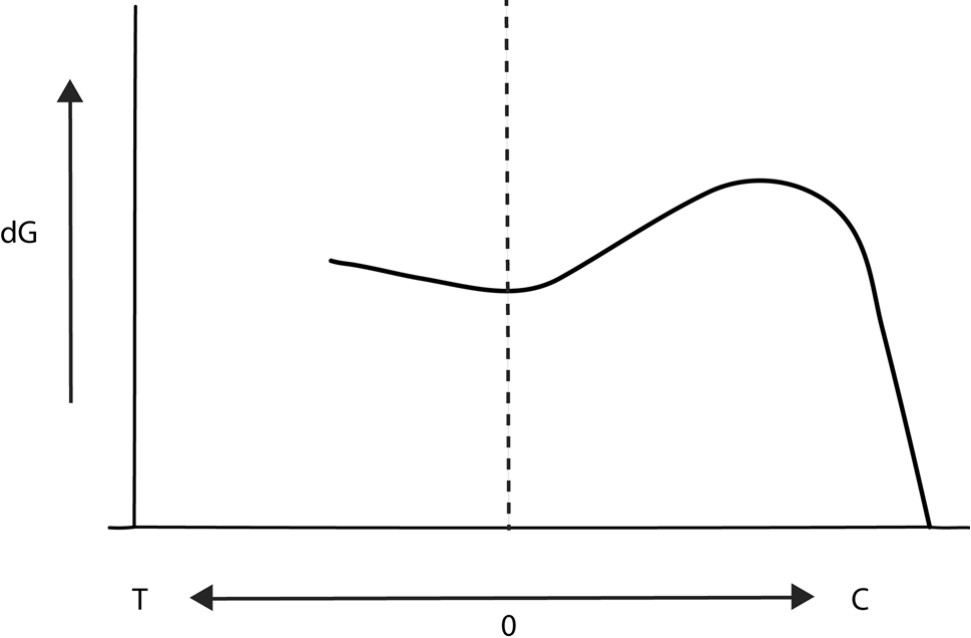
On the X-axis is tension (*T*) and compression (*C*) acting on the growth plate. On the ordinary axis is growth velocity plotted as *dG* The figure is adapted from Frost [5]

Bone remodelling continuously takes place in both children and adults. Our present understanding is still based on the concept described in Julian Wolff’s monograph on bone transformation from 1892. The observation that bone changes its external shape and internal trabecular architecture in response to the forces acting on it is commonly referred to as Wolff’s law. This is a concept for modelling and remodelling of bones to adapt to their mechanical environment. Longitudinal bone growth in the immature bone is furthermore influenced by mechanical load and this is believed to be controlled by a concept labelled the Hueter–Volkmann law. Mechanical manipulation of growth was first reported by Carl Hueter in 1862 who reported on the treatment of children with clubfeet. Through manipulation of the bones the shape could be changed during growth. Volkmann describes that changes in compressive forces on a physis would lead to different growth patterns. The concept therefore states that compressive forces acting on the physis lead to a reduction in growth velocity and that reduced load increases growth rate. These observations by Hueter and Volkmann have contributed heavily to the development of techniques to manipulate physeal growth in modern paediatric orthopaedic treatment. Frost later proposed a concept describing that chondral modelling has one response in relation to physiological loading of the growth plate [6]. Both higher or lower physiological load leads to stimulation of growth at the physis (Fig. 1).

If load of the physis is outside the physiological range, a different response is being elucidated with inhibition of growth at the physis. Sustained stress on the growth plate apparently affects growth rate in a linear fashion. These findings take into account both tensile and compressive forces, with tensile forces increasing growth rate and compression decreasing activity in the growth plate. The effect on the growth plate by dynamic load is under debate as growth rate has been reported as either being decreased or unaffected [7]. In gymnasts, abnormal ulnar-radial length differences have been reported, which is believed to be caused by great stresses placed on the forearms during gymnastics training.

## Methods and techniques for guiding growth in bones

Performing a surgical physeal arrest using permanent or potentially reversible techniques may avoid the need for osteotomies in treatment of deformities in growing children. The overall problem with performing a permanent physeal arrest is the need for timing of the procedure. The technique was first introduced by Phemister in 1933 who described a technique to obtain permanent fusion of the growth plate by performing a rectangular resection of bone containing metaphysis, physis, and epiphysis with the resected area subsequently being reinserted with the ends reversed. By using this procedure, both equalising of leg length and correction of frontal plane deformities such as genu valgum could be achieved according to Phemister. This procedure has disadvantages including considerable and prolonged postoperative care. When Canale et al. introduced a minimally invasive technique using power drills to permanently destroy the growth plate it gained popularity [8]. Transphyseal screws are also being used to obtain partial or complete growth arrest, especially in Europe. Even though the physis is not destroyed directly by transphyseal screws, this technique is effectively considered a permanent fusion of the growth plate necessitating timing of the procedure [9].

One of the first attempts to perform a reversible hemiepiphysiodesis was made by Haas who inserted a wire over the physis in growing dogs leading to growth arrest of the affected physis. Because of the seemingly good results, he proceeded to performing surgery in growing children with leg length discrepancy (LLD). Even though the wire did inhibit growth he also reported on problems with wire breakage [10]. Haas continued to work with the wire technique, but also adapted the use of staples to inhibit the growing physis.

**Fig. 2:**
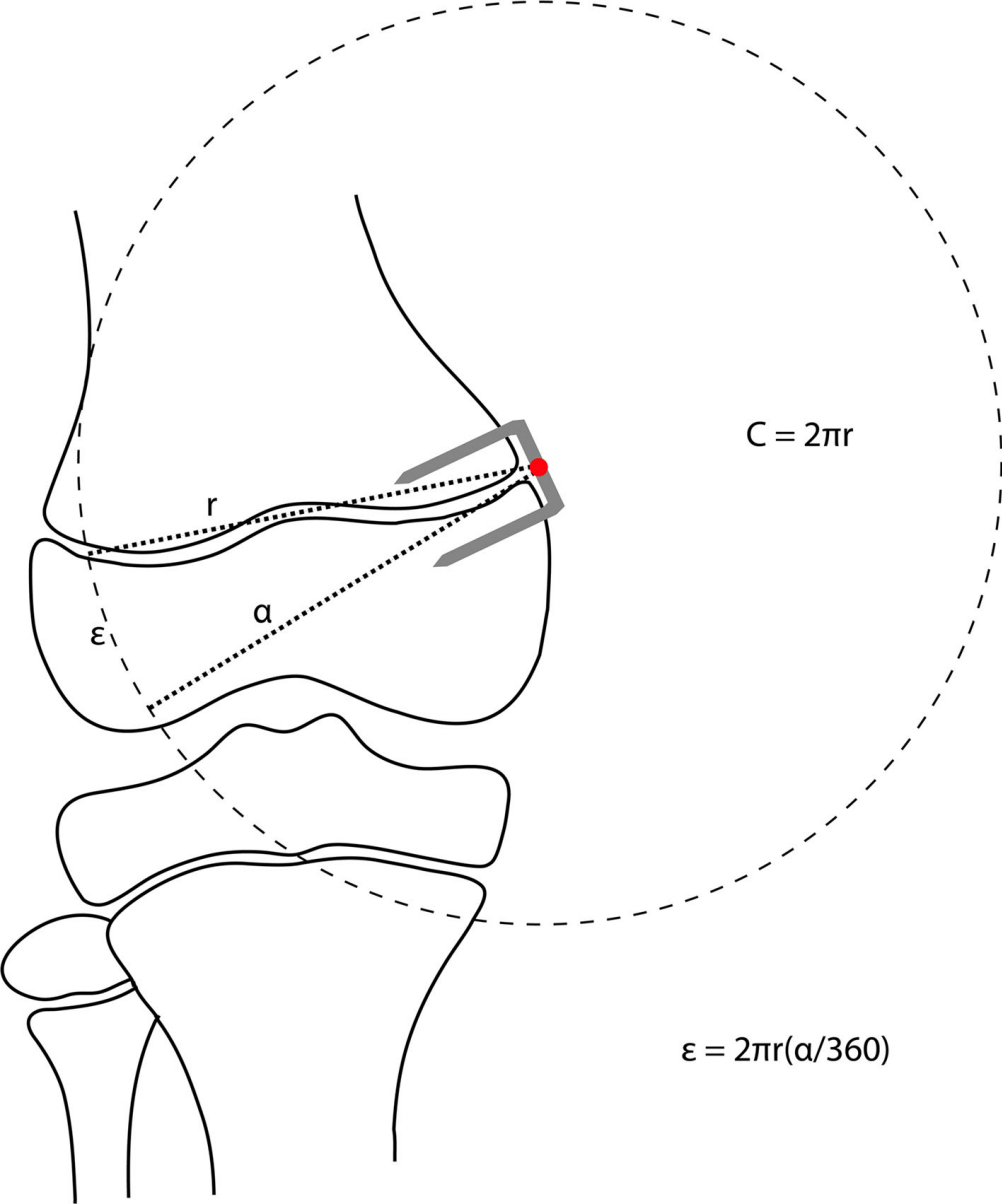
The theory behind hemiepiphysiodesis using staples The figure is adapted from Bowen et al. [11]

In 1949, Blount and Clarke reported on stapling of the epiphyseal plate as a method to correct both angular deformities and LLD [11]. They stated that implants should not be left in place for >2 years because of the risk of premature closure of the physis. They based this statement on a personal, non-published communication with Phemister. By performing partial epiphysiodesis, gradual correction of the angulating deformity was achieved. Bowen et al. described this theory using a graphic representation in 1985 (Fig. 2) [12].

Stapling is now an established procedure that has been used over the past 6 decades. Several papers have addressed the outcome regarding correction of deformities, rebound growth, and hardware failure [13, 14]. Satisfying results with correction of the deformity are reported in 80–85% of cases. Implant failure with staple extrusion or breakage is observed in approximately 10% of the treated physes. Rebound growth is believed to be more likely in children with remaining growth and slight overcorrection is recommended in children who have not reached skeletal maturity when the deformity is corrected. Because of the fear of premature closure of the growth plate, it was believed that staple correction for angular deformities should be reserved for older children close to skeletal maturity. A more recent study, however, has demonstrated that stapling can also be performed safely in younger children [15]. The actual biological response in the growth plate treated with hemiepiphysiodesis is only partially understood. Staples produce compression over the growth plate which in theory may alter the growth plate permanently. One clinical study demonstrated loss of columnar arrangement of chondrocytes in biopsies from stapled physes from children one year after surgery. The effects have been studied in more detail in animal studies. Karbowski et al. used skeletally immature pigs that had the medial proximal tibia stapled [16]. Histologically they found that the normal columnar pattern of chondrocytes in the growth plate was completely abolished in large areas with severe disturbance of the endochondral ossification. Another experimental study could detect metabolic alterations within a few days after surgery but disturbed mineralisation of the growth plate was only seen after prolonged stapling of the growth plate. It has been reported that overall leg length will change as a result of hemiepiphysiodesis although it is unclear if this has clinical relevance.

## Tension band plating

**Fig. 3:**
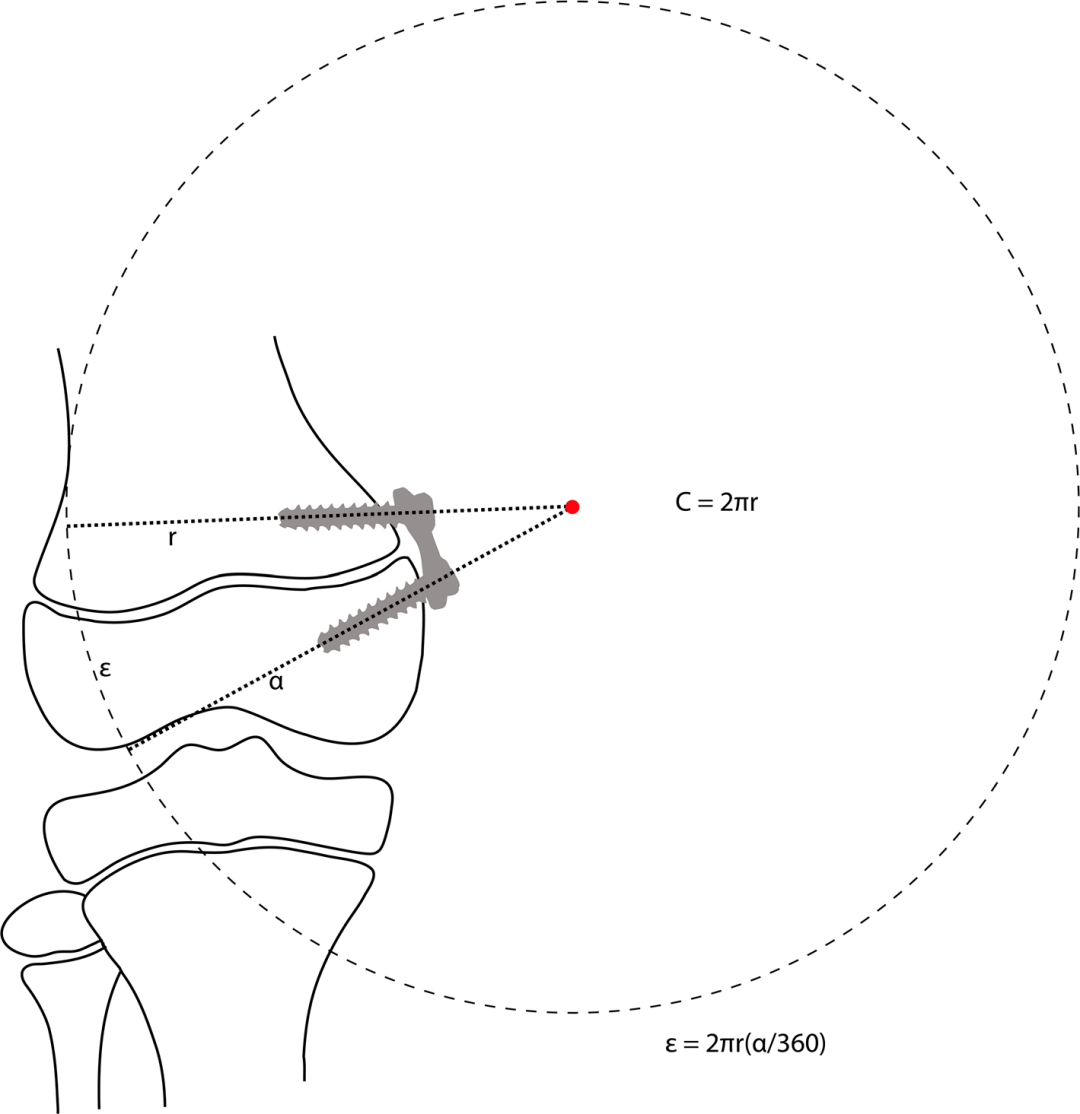
The theory behind hemiepiphysiodesis using tension band plating [2]

This is a new technique developed to address some of the complications associated with the use of staples. It was first described by Stevens who developed the eight-plate implants (Orthofix; McKinney, TX, USA). These implants have been advocated to avoid compression of the growth plate and to reduce mechanical failures [2]. The tension band plating system consists of a plate which is fixed by one screw on each side of the growth plate. The screws are not rigidly fixed in the plate and can angulate progressively as the deformity is corrected. By using tension band plating implants for hemiepiphysiodesis, the fulcrum for growth is in theory moved outside the physis (Fig. 3) compared to stapling where the fulcrum is located at the crossbar of the staple (Fig. 2).

This displacement of the fulcrum for growth around the physis would in theory lead to a different load of the physis resulting in a faster correction of the deformity [2, 12]. The tension band plating technique appears to be safe and an approximately 30% faster correction rate was reported in the first published study on its use [2]. Since then a number of publications have focused on the use of tension band plating with all studies but one being retrospective. Overall results seem satisfying and the tension band plating technique has now partly replaced stapling. The only prospective study published so far comparing staples and tension band plating (non-randomised) was unable to detect any difference between the treatments [17]. Complications with the tension band plating technique reported so far have been very few. There have been reports on hardware failure, which children suffering from pathological physis such as Blount’s disease seem to be more likely to experience or extrusion of implants during treatment [18]. These complications have since led companies to develop solid screws that can be used in patients with high risk for hardware failure.

These are specifically recommended for treatment of obese children with Blount’s disease. A number of experimental animal studies have been published on the use of the tension band plating technique. In one animal study, rabbit hind limbs were allocated to either lateral stapling, lateral tension band plating, sham surgery or no surgery. Staple migration was observed after 2 weeks of treatment. No results from the histological analysis were reported apart from the growth plate appearing disturbed between the staple legs. Interestingly, staple hemiepiphysiodesis produced a larger valgus deformity when compared to the tension band plating technique. A similar result was observed in another study on rabbits but better grasp in bone was reported for the implants used for tension band plating. In their histological analysis, cellular disorganisation was reported around implants in 9 preparations from a total of 34 femurs. No differences were found between implant types and implant related injuries to the growth plate were not observed. Two porcine animal studies compared stapling with tension band plating in paired setups similar to the study presented were also reported [19, 20]. Medial hemiepiphysiodesis induced a varus deformity using both techniques but the angulation created was larger using the tension band plating technique. Signs of early loosening of staples as well as failure of implanted staples were reported. No histological analysis was included in these studies. A recent study investigated the effect of screw length on the rate of creation of the deformity but found no difference between short and long screws. However, guided growth using the tension band plating technique was found to be more efficient overall than staples [21].

Documentation of safety and efficiency of reversible temporary epiphysiodesis is limited to retrospective clinical papers and only one experimental study. One study reported that temporary epiphysiodesis with staples in the distal femur seems to be a safe practice but staples placed bilaterally over the proximal tibia risked inducing an angulating deformity [22]. Loosening of staples overall appears to be a problem. The effect of bilateral stapling of the proximal tibia has been investigated in an animal study on rabbits. In 6 out of 10 animals that had irreversible growth arrest induced, the growth plate still appeared to be in continuity and no bony bridges were seen. Prolonged temporary epiphysiodesis using implants can therefore, in theory, lead to permanent growth arrest. In clinical practice most surgeons stick to the 2-year rule that states it is safe to leave staples over a physis for two years in a growing child. This rule originates from a 1949 paper by Blount et al. who cites a personal non-published communication with Phemister [11].

In a previous experimental study on rabbits, magnetic resonance imaging was used to investigate the effect of epiphysiodesis on the growth plate and surrounding bone. We performed an animal experimental study suggesting that temporary epiphysiodesis using tension band plating implants was feasible [23]. Clinical papers have now emphasised the problems arising with the clinical use of this technique which include failure of epiphysiodesis and problems with uncontrolled growth [24, 25].

## Permanent epiphysiodesis

As alternatives to the Phemister method, some experimental methods have been described that include the use of cytotoxic agents and other implants that cause growth inhibition; however, none of them are in active clinical use, but represent potential options.

Percutaneous methods allow a reduced hospital stay, reduced operative time, less postoperative pain, and a more cosmetic surgical scar. Moreover, a shorter rehabilitation period compared to the Phemister method is expected. A percutaneous technique should be the technique of choice for an epiphysiodesis of the growth plate for leg length discrepancies between 2 and 5 cm.

Currently used techniques for epiphysiodesis represent a potential risk for failure and complications. As the growth plates are not perfectly flat, there are significant technical challenges to ensure that the tip of the tool is in the plate and that it stays in place throughout the procedure. Complications such as breaching the anterior or posterior cortex of the femur have potentially serious consequences with a risk of vascular and/or nerve injury. Further damage to the metaphyseal region of the bone may be incurred through excessive curettage and drilling. Therefore, there is a need for a reliable and precise procedure which overcomes the complications.

## Radiofrequency ablation (RFA)

In 1992, Rosenthal et al. first described RFA of osteoid osteoma, and since then it has become the standard treatment. It has been shown to be a reliable technique for creating thermally induced coagulation necrosis. Several studies have shown the usefulness of RFA as a minimally invasive image-guided technique [26]. Its advantages over surgical treatment are numerous, mainly the feasibility in an outpatient setting, a low complication rate, and short recovery time. Ghanem et al. proved that growth arrest can be achieved by radiofrequency coagulation of the physis by studying 60 New Zealand white rabbits [27]. In a prospective study, RFA epiphysiodesis has been proved to be effective and safe in a porcine model [28, 29].

However, pre-clinical studies are needed to determine the ideal parameters to achieve spontaneous irreversible and temporary growth arrest in humans [28, 30].

## Summary

Guided growth is a well-proven concept for correcting deformities in children. Mechanical stimuli such as tension and compression influence the growth rate, but exactly how growth is regulated is poorly understood. Excessive compression slows down growth and leads to a disorganised growth plate. Traditionally, staples have been used, but tension band devices are now increasingly being used due to low complication rates and ease of use. After removal of the mechanical compression, rebound growth may occur and it should be taken into consideration when planning removal of implants for epiphysiodesis. For LLD, reversible epiphysiodesis is a tempting option as it solves some of the problems of timing with irreversible epiphysiodesis where predicted growth has to be estimated. However, the technique has been criticised for poor efficiency and for the inherent risk of creating secondary deformities as growth is not inhibited equally throughout the growth plate. For irreversible epiphysiodesis, Canale’s percutaneous drilling technique produces reliable results, but may in the future be replaced by other less invasive techniques such as RFA. With the introduction of reliable expandable intramedullary nails the traditional indication for epiphysiodesis of 2–5 cm LLD may be changing, but is unlikely to fully disappear as it can also be used in conditions with abnormal high growth rates such as Marfan syndrome.
